# Relevance of measurement of bêta-2-microglobulin in schizophrenia

**DOI:** 10.1192/j.eurpsy.2024.294

**Published:** 2024-08-27

**Authors:** A. Aissa, A. B. C. Arij, S. Jedda, F. Askri, R. Jomli, H. Abaza

**Affiliations:** ^1^Psychiatry A, Razi Hospital, Manouba; ^2^Psychiatry, Faculty of Medicine of Tunis; ^3^Human genetic lab, Salah Azaiez Institute, Tunis; ^4^Clinical biology, Razi Hospital, Manouba, Tunisia

## Abstract

**Introduction:**

There are several arguments supporting the inflammatory hypothesis in schizophrenia (SCZ). Among the inflammatory markers, beta-2- microglobulin (β2M) is associated with abnormalities in neurogenesis and cognitive impairment described in (SCZ).

**Objectives:**

The objectives of our study were to evaluate the level of β2M in a group of patients compared with a control group and to investigate the sociodemographic, clinical, and environmental factors associated with elevated β2M levels

**Methods:**

We conducted a cross-sectional in outpatients with SCZ. We collected patients sociodemographic, environmental, and clinical data. We assessed psychopathology with the PANSS. We measured serum β2M concentration.

**Results:**

We included 30 patients with SCZ compared with 20 controls. Patients mean age was 40,23±10,66. The mean level of β2M was 1,98 ± 0.42 mg/L for patients and 1.65±0.56 mg/L for control group. The difference was significant between the patient group and the control group (p&lt;10-3). No environmental or clinical factors have been associated with β2M levels other than smoking status (p=0.046).

**Conclusions:**

Further research with larger samples investigating the different stages of SCZ especially early psychosis would be needed to confirm the relevance of this biomarker in SCZ.

**Disclosure of Interest:**

None Declared

